# Ankle joint distraction arthroplasty for severe ankle arthritis

**DOI:** 10.1186/s12891-017-1457-9

**Published:** 2017-02-28

**Authors:** Yang Xu, Yuan Zhu, Xiang-yang Xu

**Affiliations:** 1 Department of Orthopedics, Shanghai Ruijin Hospital, Shanghai Jiaotong University School of Medicine, Shanghai, 200025 China; 2 Shanghai Institute of Traumatology and Orthopedics, Shanghai, China

**Keywords:** Ankle joint, Distraction, Arthroplasty

## Abstract

**Background:**

Ankle distraction arthroplasty is one option for the treatment of severe ankle arthritis in young patients. The outcomes and factors predicting success in distraction arthroplasty are poorly understood.

**Methods:**

From January 2011 to May 2015, 16 patients who had undergone ankle distraction arthroplasty for ankle arthritis were operated, including six males and ten females. All patients were available for analysis. The main outcome measurements included joint space on weight bearing radiographs, AOFAS-AH scores (American Orthopaedic Foot & Ankle Society ankle-hindfoot score), VAS scores and SF-36 scores.

**Results:**

All 16 patients were followed for a mean follow-up of 40.9 ± 14.7 months (range, 17–67 months). Fourteen of the 16 patients still had their native ankle joints. One patient had undergone ankle arthrodesis 1 year after the operation and one patient had converted to spontaneous ankle fusion at the 3 years follow-up postoperative. The VAS score improved from 5.9 ± 0.8 to 3.7 ± 2.2 (*p* = 0.0028). The mean AOFAS-AH score improved from 41.9 ± 7.2 preoperatively to 68.1 ± 20.0 postoperatively (*p* = 0.001). The mean SF-36 score improved from 43.1 ± 7.6 preoperatively to 62.7 ± 18.8 postoperatively (*p* = 0.002). A weight-bearing ankle space larger than 3 mm at 1 year following distraction is a positive predictive factor.

**Conclusions:**

In this study, the treatment of ankle motion distraction for end stage ankle arthritis showed benefit in 9/16 (56.25%) patients at 41 months. It is a promising method for young patients with severe ankle arthritis.

## Background

Ankle joint distraction arthroplasty is a promising treatment method for ankle arthritis but the outcomes of distraction arthroplasty are poorly understood. It is an effective treatment for young patients with end-stage ankle arthritis, providing the benefits of pain reduction and functional improvement. In addition to ankle osteoarthritis, young patients with rheumatoid arthritis of the ankle joint also achieved good results [1]. The purpose of ankle distraction is to allow for intermittent intra-articular fluid pressures, the relief of mechanical stress on the cartilage and sustained, diminished subchondral sclerosis following the procedure [2, 3]. All of the mechanisms mentioned above are of benefit for cartilage repair and the reduction of cartilage damage. However, the current literature lacks good studies on distraction arthroplasty [4].

On the basis of the available clinical evidence, we conducted this study and hypothesized that ankle motion distraction would alleviate symptoms, improve ankle function and delay the time to ankle arthroplasty or arthrodesis while maintaining the joint space for a long period of time.

## Methods

Approval for this retrospective study was obtained from our hospital’s ethics committee.

The inclusion criteria for patients included the following: (1) symptomatic isolated, unilateral severe ankle osteoarthritis; (2) younger than 60 years old; (3) painful ankle arthritis with more than 1 year of conservative treatment including ankle debridement and medication.

The exclusion criteria included the following: (1) acute or chronic infection; (2) older than 60 years old; (3) poor general health condition; (4) patients who had ankle surgery like supramalleolar osteotomy during ankle distraction or during follow-up. Because we were not sure whether or not the outcomes were due to ankle distraction.

All the patients were followed and none dropped out. Since we decided to analyze the outcomes of ankle distraction, all patients were required to come to our clinic during the last 3 months before submission of this paper. We got 15 patients’ X-rays and latest information during this period. The other patient was operated 6 years ago. The last X-ray of her was 4 years postoperative. But we still keep contact with her via telephone.

### Preoperative preparation

All patients were evaluated and their complete histories were obtained. Preoperative routine weight bearing radiographs were performed, including anteroposterior, mortise and lateral views of the ankle joint. The hindfoot alignment view was also made to help in the evaluation of hindfoot alignment. The hindfoot alignment view we used was recommended by Saltzman and el-Khoury [5]. CT and MRI scans were obtained before the surgery and at the follow-up visits to evaluate the condition of the ankle joint cartilage and subchondral bone.

Before the surgery, the patients were examined and circumferential external fixators were constructed based on the size and length of each patient’s leg and foot. All VAS scores, AOFAS-AH scores (American Orthopaedic Foot & Ankle Society ankle-hindfoot score) and SF-36 scores were collected preoperatively as customary at our center. The VAS score was used to evaluate the degree of pain. The AOFAS-AH score and SF-36 score were used to evaluate ankle function.

### Surgical technique

The patient was placed supine on the operating table. First, a complete cheilectomy was performed to clear the osseous impingement. We did adequate resection of anterior tibial osteophytes to make sure that when dorsiflexing the ankle joint, there was no impingement. Intraoperative lateral fluoroscopic images were used to assess the resection. All visual osteophytes around ankle joint were completely resected. Osteophytes around the ankle joint were removed through an anterior open incision or a lateral or posterior incision, if necessary.

**Fig. 1:**
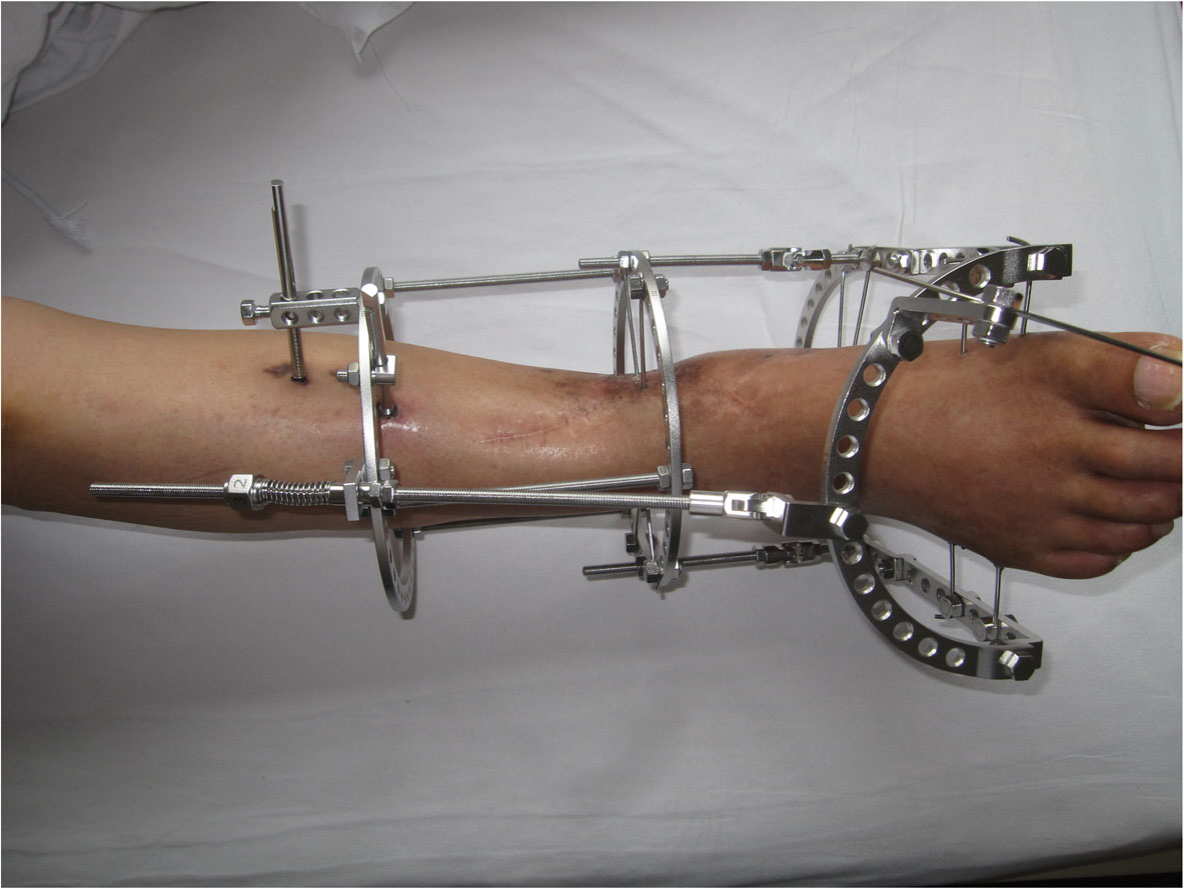
Photograph of ankle distractor

The external fixator was applied in a standard fashion. The external fixator included two tibial rings and one podalic ring. The whole external fixator was constructed and sterilized before the surgery. A K-wire was inserted into the axis of the ankle motion from the tip of the medial malleolus to the tip of the lateral malleolus temporarily and the external fixation frame was installed to make sure that the axis of the ankle motion was consistent with the axis of the external fixation frame motion. This was the most important step. The frame was fixed with K-wires or half pins if necessary. Each tibial ring was fixed with two crossed K-wires. If the patient had osteoporosis, half pins were utilized to help stabilize the external fixator. Foot fixation included four 2.0 mm tensioned K-wires. Two crossed K-wires were inserted through the calcaneum, and two K-wires were drilled through the metatarsals and midfoot to fix the forefoot and midfoot. C-arm fluoroscopy was used to ensure ideal wire placement. After all wires and pins were placed correctly, the temporary K-wire inserted into the axis of ankle motion was removed (Fig. 1).

### Postoperative management

Distraction by 1 mm per day was performed after surgery and was assessed by radiography. Full weight bearing was permitted 2 weeks postoperatively. We required the patients walk with the fixators 2 weeks postoperatively, and the motion exercises were performed from the 2nd week to the 12th week. After the surgery, every patient underwent routine pinhole nursing every day. The distractor was removed 3 months after surgery. Each patient was required to come to the outpatient department every year to evaluate the function of the ankle and to take radiographs. Unfortunately, not every patient complied with this demand to visit every year. The postoperative VAS, AOFAS-AH and SF-36 scores analyzed in this study were the latest information collected by telephone or outpatient visit.

### Statistical methods

The significance level was set at α = 0.05. All data analyses were performed with SAS software version 8.1 (SAS Institute, Inc, Cary, NC). The results were presented as the mean and standard deviation. The paired Student’s *t*-test was used to compare the preoperative and postoperative radiographic measurements and ankle function scores.

## Results

From January 2011 to May 2015, 126 patients were screened and 17 patients entered into this study. One patient had supramalleolar osteotomy during ankle joint distraction and was excluded. In this case, the patients had a history of severe Pilon fracture and had severe posttraumatic ankle varus deformity and ankle arthritis. So we did a supramalleolar osteotomy to realign the ankle alignment. The remaining 16 patients (6 males and 10 females) who had undergone ankle distraction arthroplasty met the criteria. Table 1 shows the patient’s detailed information. The average patient age at surgery was 30.3 ± 14.3 years old (range, 14 to 60 years). Among these, 13 patients had a history of ankle fracture or Pilon fracture. One patient had a history of an old talus fracture. One patient had a history of ankle sprain 10 years prior to the study, and the other one had no prior history of ankle injury. One patient also underwent the fibula reduction with distraction at the same time. Debridement and removal of osteophytes were performed in 6 patients. The Achilles tendons of two patients were lengthened at the time of operation with percutaneous Hoke method. The mean follow-up time was 40.9 ± 14.7 months (range, 17 to 67 months) (Table 1).

**Table 1:** Outcomes of ankle distraction for ankle arthritis

Patient no.	Left/Right	History	Time of follow-up (month)	VAS preop	AOFAS preop	SF-36 preop	VAS postop	AOFAS postop	SF-36 postop	Clinical outcome
1	L	ankle fracture	17	6	36	33.53	3	78	57.74	good
2	R	ankle fracture	24	6	42	44.36	1	81	77.61	good
3	L	ankle fracture	44	7	29	66.22	2	88	78.02	good
4	R	-	66	6	39	42.05	6	39	41.94	fair
5	R	ankle fracture	63	5	48	41.12	6	46	41.31	fair
6	L	ankle fracture	36	7	51	50.35	5	58	67.44	good
7	L	ankle sprain	29	5	36	39.97	5	41	41.36	fair
8	R	ankle fracture	67	6	42	43.34	4	60	59.72	good
9	R	ankle fracture	32	5	31	37.09	0	100	89.64	excellent
10	L	ankle fracture	50	6	51	42.41	6	57	50.13	fair
11	R	talus fracture	37	-	-	-	-	-	-	failed
12	R	ankle fracture	32	7	44	43.01	7	56	43.9	fair
13	L	ankle fracture	32	7	52	40.78	2	90	87.55	excellent
14	L	ankle fracture	39	5	44	42.84	4	68	51.28	good
15	R	pilon fracture	37	-	-	-	-	-	-	failed
16	L	ankle fracture	49	5	42	48.72	1	91	90.06	excellent

During the follow-up, one patient had talonavicular joint arthritis 3 years after the operation, but the patient was very satisfied with the ankle joint function although the ankle joint motion was comparatively less than normal. One patient had calcaneal osteotomy due to hindfoot valgus alignment. Five patients had their ankle space return to the same as or narrower than their preoperative joint space. One patient’s ankle joint converted to spontaneous fusion at the 3-year follow-up, and this patient had pain relief. One of the patients with severe Pilon fracture ultimately transferred to ankle arthrodesis in 2015, 2 years after ankle distraction arthroplasty. The other 9 patients remained at a normal or wider joint space at the latest follow-up and had positive outcomes. A total of 14 patients had native ankle joints at the latest follow-up. Table 2 shows that the mean VAS score improved from 5.9 ± 0.8, preoperatively, to 3.7 ± 2.2, postoperatively (*p* = 0.0028). The mean AOFAS-AH score improved from 41.9 ± 7.2, preoperatively, to 68.1 ± 20.0, postoperatively (*p* = 0.001). The mean SF-36 score improved from 43.1 ± 7.6, preoperatively, to 62.7 ± 18.8, postoperatively (*p* = 0.002) (Table 2). In this study, 3 patients rated their outcomes as “excellent”, and a “good” outcome was observed in 6 patients. Five patients rated their results as “fair” because of the consistent discomfort. On the other hand, 2 distraction surgeries failed, with one patient undergoing ankle arthrodesis and another with the spontaneous fusion of the ankle joint. Only two patients had pin-site infection. In this study, the use of ankle motion distraction for the treatment of severe ankle arthritis showed benefit in 9/16 patients at 41 months. The mean preoperative ankle space was 1.9 mm (range, 1.6 to 2.4 mm), the mean ankle space during distraction was 6.2 (range, 5 to 8.83 mm), and the mean ankle space of last follow-up was 4.1 mm (range, 2.2 to 5.48 mm). We noticed that patients with ankle space larger than 3 mm tended to achieve better outcomes. A weight-bearing ankle space larger than 3 mm at 1 year following distraction is a positive predictive factor.

**Table 2:** Functional outcomes after ankle distraction

	Preop	Postop	*P* value
VAS	5.9 ± 0.8	3.7 ± 2.2	0.0028
AOFAS-AH	41.9 ± 7.2	68.1 ± 20.0	0.001
SF-36	43.1 ± 7.6	62.7 ± 18.8	0.002

The average preoperative and postoperative ankle dorsiflexion ranges were 6.5° (range, 0 to 12°) and 8° (range, 0 to 15°) (*p* > 0.05). The average preoperative and postoperative ankle plantarflexion ranges were 33.2° (range, 20° to 45°) and 36° (range, 20° to 50°) (*p* < 0.05). The average preoperative and postoperative ankle motion ranges were 39.7° (range, 20° to 55°) and 44° (range, 25° to 60°) (*p* < 0.05).

**Fig. 2:**
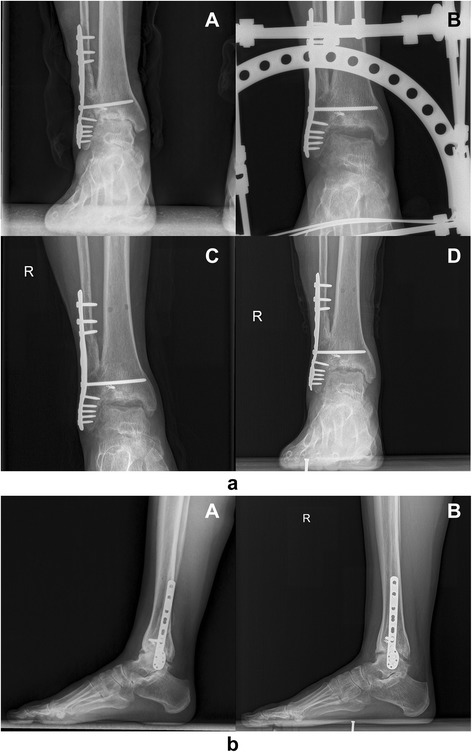
**a** radiographs of a female with severe ankle arthritis. *a* preoperative ankle joint. *b* ankle joint during ankle distraction. *c* ankle joint after removal of external fixation. *d* 1 year postoperative. **b** lateral radiographs of a female with severe ankle arthritis. *a* lateral view of the same patient before operation. *b* lateral view of the same patient at 1 year after operation

Fisher’s Exact Test was used to analyze whether gender was one of the prognostic factors. And the *P* value was larger than 0.05, which showed gender had no effect on prognosis (Fig. 2a) (b).

## Discussion

Posttraumatic arthritis is the most common form of ankle arthritis. Ankle fractures can cause altered biomechanics and structural changes even with adequate open reduction and internal fixation [3]. Ligamentous injury is another important cause of posttraumatic ankle arthritis.

The goals of treatment for ankle osteoarthritis are reducing pain and restoring as much ankle function as possible [6]. Conservative treatments for early stage ankle arthritis include lifestyle adaption, oral medicine such as NSAIDS. Joint preservation surgical treatments include ankle debridement, osteotomy and joint distraction. Joint sacrificing treatments include arthrodesis and joint replacement. Ankle distraction, arthrodesis and replacement are indicated for end-stage ankle osteoarthritis. Arthrodesis has the potential risk of adjacent joint arthritis, continuous pain, nonunion and decreased function [7–10]. Ankle replacement is associated with the risk of device-related infection and major revision surgery [11, 12]. For ankle arthritis, supramalleolar osteotomy is indicated for early ankle arthritis with partially preserved ankle joint surface, ankle frontal alignment deformities and retaining the motion of the ankle [13–15]. Ankle arthritis with subsequent subtalar arthritis is a good indication of tibiotalocalcaneal arthrodesis [16].

However, for relatively young cases, ankle distraction may be a better choice. The mechanisms of ankle distraction may attribute to the relief of mechanical stress on the cartilage, the formation of intermittent fluid pressure that is beneficial to the nutrition of cartilage and decreasing subcohondral sclerosis [2].

Van valburg et al. recommended that distraction should be carried out over a distance of 5 mm on weight-bearing X-rays [17]. Fragomen et al. came to the conclusion through cadaveric experiments that a 5.8 mm distraction gap on X-ray was needed under weight-bearing conditions, while a 7.0 mm distraction gap on X-ray was needed under non-weight-bearing conditions [18]. Few studies emphasize the ankle space change after ankle distraction. In our study, we noticed that there was a reduction of ankle space after taking off the external fixation. However, if the ankle space could maintain a normal or larger than normal space 1 year postoperatively, the joint space may continue to stay in this condition long term.

The fixator used in the distraction arthroplasty can be fixed or mobilizable. In our study, all patients’ fixators were mobilizable. In a prospective randomized controlled trial, Saltzman et al. reported that outcomes after motion distraction were significantly better than those using fixed distraction [19]. Moreover, in their subsequent study, Nguyen et al. reported that AOS scores at 2 years after distraction and age at the time of surgery were the most important predictive factors of ankle survival. They thought that those with an AOS score smaller than 42 and older patients tended to achieve better outcomes [20]. They questioned whether motion distraction was superior to fixed distraction because of the limited number of patients and the fixed group regaining motion over time. However, in their studies, there was no description of the distraction distance before and after the operation and no mention of joint space improvement. Marijnissen et al. found that the female gender is a predictor of failure of distraction arthroplasty [21]. Female patients had a higher likelihood of failure than male patients, which is a finding similar to that found in hip distraction. In our study, we found that it is the postoperative ankle space that is the most important predictive factor. If the ankle space remained larger than 3 mm at the 1-year postoperative follow-up under the weight-bearing situation, the outcomes tend to be better than those with an ankle space smaller than 3 mm. The weight-baring ankle spaces of those who achieved pain relief or clinical symptoms improvement were larger than 3 mm.

In this study, six patients had preceding arthroscopic debridement or debridement with open incision. In previous studies, ankle distraction had a statistically significant better result than debridement, and it was demonstrated that the improvement in the clinical outcome after ankle distraction was not due to debridement [2, 22].

The most common complication of distraction is pin-site infection. Others like osteomyelitis, deep vein thrombosis and K-wire breakage were reported [23]. In our study only two patients had pin-site infection, which was resolved by oral antibiotics and routinely dressing change.

There are several limitations of this study. First, it was a retrospective study of a case series, and there was no control group. Thus, we were not sure whether there was a placebo effect. Six patients underwent ankle joint debridement and osteophyte excision, and one of the patients also underwent a reduction of the fibula. We were not sure how much these additional procedures could have influenced the outcomes, although a previous study has demonstrated that the clinical outcome improvement is not due to debridement [2]. The articular surface of severe ankle arthritis patients is blurred, which makes it hard to measure the joint space. Although we tried to measure the space as many times as possible, it may not represent the real ankle space. Thus, the ankle space during long-term visits may not represent the real average. Since we noticed an ankle fusion during follow-up, the possibility of spontaneous ankle fusion should not be neglected even though the patient experienced pain relief.

## Conclusion

In this study, the treatment of ankle motion distraction for severe ankle arthritis showed benefit in 9/16 (56.25%) patients at 41 months. It is a promising method for young patients with severe ankle arthritis.

## Abbreviations

AOFAS-AH: American Orthopaedic Foot & Ankle Society ankle-hindfoot
AOS: Ankle Osteoarthritis scale
SF-36: Short form-36
VAS: Visual analogue scale
